# K469E polymorphism of the intercellular adhesion molecule-1 gene in Egyptians with coronary heart disease

**DOI:** 10.4103/0256-4947.71061

**Published:** 2010

**Authors:** Amal A. Mohamed, Laila Rashed, Hoda Amin, Manal Abu-Farha, Soheir Abu El Fadl, Sameh Pakhoum

**Affiliations:** a From the Department of Clinical and Chemical Pathology, Faculty of Medicine, Cairo University, Egypt; b From the Department of Biochemistry, Faculty of Medicine, Cairo University, Egypt; c From the Department of General Medicine, Faculty of Medicine, Cairo University, Egypt; d From the Department of Cardiology, Faculty of Medicine, Cairo University, Egypt

## Abstract

**BACKGROUND AND OBJECTIVES:**

The initial step in atherosclerosis is the adhesion of leukocytes to activated endothelial cells mediated by intercellular adhesion molecule-1 (ICAM-1). This study aimed to investigate the association of K469E polymorphism of the *ICAM-1* gene and soluble ICAM-1 (sICAM-1) serum level with coronary heart disease (CHD) in Egyptian subjects.

**PATIENTS AND METHODS:**

Using a case-control design, we studied 100 patients with CHD, including 73 patients with acute myocardial infarction (MI) and 27 with unstable angina (UA). The control group consisted of 50 healthy subjects with normal left ventricular function. All participants were genotyped for the ICAM-1 polymorphism by the polymerase chain reaction-restriction fragment length polymorphism (PCR-RFLP) method. Serum sICAM-1 was measured by enzyme-linked immunoassay (ELISA).

**RESULTS:**

In CHD patients, the frequencies of K genotype (KK and EK) were significantly higher when compared to controls (*P*<.001) and were associated with an increased risk of disease development (OR=3.8, 95% CI: 1.7 to 8.5; *P*=.001). K genotype frequencies in patients with MI showed no significant difference when compared to patients with UA (*P*= .121). Serum sICAM-1 levels were comparable between CHD patients and controls (*P*= .37) and between MI and UA patients (*P*=.23). There were no significant differences in sICAM-1 levels among patients with different genotypes (*P*=.532). Men presented with higher sICAM-1 levels than women (*P*=.004).

**CONCLUSION:**

ICAM-1 gene polymorphism in codon 469 is associated with a risk for CHD development in Egyptian subjects. Serum sICAM-1 is not influenced by this polymorphism and is not necessarily elevated in CHD.

Cardiovascular disease (CVD) remains the leading cause of death in the developed world. Although hyperlipidemia screening and treatment has proven to be one of the most effective tools for reducing CVD burden, it often fails to identify a substantial proportion of persons at high risk for CVD-associated events. Elevations in markers of inflammation and thrombosis, such as high sensitivity C-reactive protein, soluble intercellular adhesion molecule, homocysteine and fibrinogen, are also associated with increased CVD risk.^1^ Intercellular adhesion molecule-1 (ICAM-1) is an important inflammatory protein; it is one of the adhesion molecules that plays a key role in the recruitment of leukocytes at sites of inflammation and the tight adhesion between leukocytes and vascular endothelium. Inflammation plays a critical role in atherogenesis. The initial step in atherosclerosis is the adhesion of leukocytes to activated endothelial cells mediated by intracellular adhesion molecule-1 (ICAM-1).^2^ Soluble ICAM-1 (sICAM-1) is a circulating form of ICAM-1 and has been shown to reflect cell surface expression of ICAM-1 in human vascular endothelial cells. The main mechanism responsible for release of sICAM-1 is believed to be proteolytic cleavage of ICAM-1 from cell membranes.^3^ The *ICAM-1* gene is located on chromosome 19 p13.2-p13.3. A single-base C to T transition polymorphism, which results in an amino acid substitution from glutamine (E) to lysine (K) in the ICAM protein in exon 6 codon 469, has been found to be related to inflammatory diseases and atherosclerosis.^4^ The aim of this study was to investigate the association of K469E polymorphism of the *ICAM-1* gene and sICAM-1 serum level with coronary heart disease (CHD) in Egyptian subjects.

## PATIENTS AND METHODS

### 

The study population consisted of 100 patients with acute CHD, including 73 patients with acute myocardial infarction (MI) and 27 patients with unstable angina (UA) (mean [standard deviation] age: 52.6 [8.29] years; and male/female ratio: 87/13). They were recruited from the Department of Internal Medicine and critical care unit of Cairo University Hospitals in the period from October 2008 to June 2009. The diagnosis of CHD was based on medical history, physical examination, typical electrocardiographic changes, increases in serum cardiac enzymes activities and coronary angiography. Fifty healthy subjects with normal left ventricular function constituted the control group (mean [SD] age: 53.9 [6.2] years, and male/female ratio: 38/12). Subjects with any inflammatory condition were excluded. Blood samples were collected after a fast of 12 to 14 hours from all subjects after obtaining oral consent. Serum and EDTA samples were stored at –20°C until assay time.

Assay of serum total cholesterol, HDL and triglycerides was performed on automated analyzer, Hitachi 917; commercial kits were supplied by Roche Diagnostics (Boehringer Mannheim, GmbH D-68298, Mannheim, Germany). The LDL level was calculated using the Friedwald equation. Soluble ICAM-1 levels in serum were measured by solid-phase sandwich enzyme-linked immunoassay (ELISA) kit supplied by Thermo Scientific Life Science, USA.^5^

#### Molecular analysis of K469E polymorphism of ICAM-1 gene

DNA extraction was performed using the standard salting-out technique.^6^ Genotype analysis was performed using the polymerase chain reaction-restriction fragment length polymorphism (PCR-RFLP) method. The sense primer was 5’-GGA ACC CAT TGC CCG AGC -3’, and the antisense primer was 5’- GGT GAG GAT TGC ATT AGG TC -3’. PCR amplification of a 223-bp fragment of exon 6 was performed in 50-μL reaction mixture consisting of 67 mM Tris-HCl (pH, 8.8), 1 mM magnesium chloride, 10 mM dNTPs, 5 pmol of each primer, 100 ng of extracted DNA and 2.5 units of Taq DNA polymerase (Amersham Pharmacia Biotech.).

Thermal cycling was carried out with an initial 95°C denaturation step for 10 minutes, followed by 35 cycles of denaturation for 1 minute at 95°C, annealing for 1 minute at 58°C, extension for 1 minute at 72°C and a final extension step at 72°C for 10 minutes. The PCR product was then digested with the restriction enzyme BstU I (Amersham Pharmacia Biotech., USA) for 12 hours at 37°C. The fragment length of the KK genotype was 223 bp; the fragment lengths of the EE genotype were 136 and 87 bp; the fragment lengths of the EK genotype were 223, 136 and 87 bp. The products of the digestion process were separated by electrophoresis on a 2.5% agarose gel, followed by ethidium bromide staining.^4^

Data were statistically described in terms of mean and standard deviation (SD), frequencies (number of cases) and relative frequencies (percentages). Comparison of quantitative variables between the study groups was done using the *t* test and one-way ANOVA test for independent samples. For comparing categorical data, chi-square test was performed. Risk analysis was performed by calculating the odds ratio (OR) and the 95% confidence interval (CI). A *P* value less than .05 was considered statistically significant. All statistical calculations were done using computer programs Microsoft Excel version 7 (Microsoft Corporation, NY, USA) and SPSS (Statistical Package for the Social Science; SPSS Inc., Chicago, IL, USA) version 15 for Microsoft Windows.

## RESULTS

### 

This study included 100 CHD patients, 27 of whom had unstable angina (UA) (22 men and 5 women, mean [SD] age, 54.9 [8.7] years), and 73 patients had acute myocardial infarction (MI) (65 men and 8 women, mean [SD] age, 50.3 [7.9] years). The control group consisted of 50 healthy subjects. There were no significant differences in age and gender between CHD patients and controls. Patients with hypercholesterolemia and smokers were more frequent in the CHD group when compared with controls (**Table 1**).

**Table 1:** Characteristics of coronary heart disease (CHD) patients (n=100) and controls (n= 50).

Variable	CHD patients	Controls	*P*
Age (years)^a^	52.6 (8.29)	53.9 (6.2)	.34
Gender (male /female)	87 /13	38 /12	.23
Hypertension (%)	71	68	.70
Smoking (%)	65	39	.002
Diabetes mellitus (%)	30	26	.61
Hypercholesterolemia (%)	79	52	.001
ICAM level (pg/mL)^a^	838.79 (153.93)	802.55 (144.68)	.37

a Values are mean (standard deviation).

#### Distribution of allele and genotype frequencies

The genotype frequencies were 43% EE, 37% EK and 20% KK in patients with CHD, thus differing from those in the control subjects, which were 74% EE, 22% EK and 4% KK. The genotype frequencies were 38.3% EE, 38.3% EK and 23.3% KK in patients with MI and 55.6% EE, 33.3% EK and 11.1% KK in patients with UA, showing no significant difference (**Table 2**).

**Table 2:** Frequencies of the K469E variants of *ICAM-1* gene in CHD (n=100), unstable angina (UA) (n=27), myocardial infarction (MI) (n=73) and controls (n= 50).

	CHD	UA	MI	Controls
ICAM-1 Genotype n (%)				
EE	43 (43)	15 (55.6)	28 (38.33)	37 (74)
EK	37 (37)^a^	9 (33.3)^a^	28 (38.33)^a^	11 (22)
KK	20 (20)^a^	3 (11.1)^a^	17 (23.3)^a^	2 (4.0)
KK+EK	57(57)^a^	12 (44.4)^a^	45 (61.65)^a^	13 (26.0)
ICAM-1 allele n (%)				
E	123 (61.5)	39 (72.2)	84 (57.5)	85 (85)
K	77 (38.5)^a^	15 (27.8)^a^	62 (42.5)^a^	15 (15)

a Significantly increased as compared to controls.

Analysis of the genotype with respect to dominant and additive effects of the K allele showed that individuals with K allele (KK and EK) had a significantly increased risk of CHD (**Table 3**).

**Table 3:** Odds ratios for CHD patients heterozygous or homozygous for the -469 K variant.

Genotype	Odds ratio (95% CI)	*P*
EK	2.9 (1.2-7.8)	.014
KK	8.6 (1.8-57.2)	.003
KK+EK	3.8 (1.7-8.5)	.001

#### Serum sICAM-1

sICAM-1 serum levels showed no significant difference between CHD patients (mean [SD], 838.79 (153.93)pg/mL) and control subjects (mean [SD], 802.55 (144.68)pg/mL) (*P*=.37). In the CHD group, sICAM-1 levels were comparable between MI (mean [SD], 828.17 (158.72)pg/mL) and UA (mean [SD], 867.52 (138.87)pg/mL) (*P*=.23). There were no significant differences in sICAM-1 levels between patients with different genotypes; EE (mean [SD], 809.51 (132.8)pg/mL), EK (mean [SD], 816.21 (116.82)pg/mL) and KK (mean [SD], 847.23 (125.7)pg/mL) (*P*=.532). A significant difference was found between men (854.966 (153.33)pg/mL) and women (720.183 (99.36) pg/mL) (*P*=.004).

## DISCUSSION

Leukocyte adherence to the vascular endothelium is one of the earliest demonstrable events in atherosclerosis.^7^ Intercellular adhesion molecule-1 (ICAM-1) mediates the interaction of activated endothelial cells with leukocytes and plays a fundamental role in the pathogenesis of coronary atherosclerosis. ICAM-1 single-base C/T polymorphism, which determines an amino acid substitution from glutamine (E) to lysine (K) in the ICAM-1 protein in exon 6 codon 469, has been described.^8^

In the current study, ICAM-1 EK and KK variants represented 57% of our CHD patients and were associated with increased risk of disease development; OR=3.8 (95% CI: 1.7 to 8.5; ^P^=.001). This agrees with the findings of Jiang et al in German patients with CHD, who suggested an important role for ICAM-1 gene polymorphism in codon 469 in the susceptibility for CHD and MI. In Han Chinese population, Zhang et al^9^ found that the presence of KK genotype of ICAM-1 codon 469 conferred an increased risk for CHD. Also, Liu et al^4^ reported that KK homozygotes had a higher risk of re-stenosis after coronary stenting and concluded that the ICAM-1 KK genotype may serve as a predictor of in-stent re-stenosis, especially in obese and hyperlipemia patients. Similarly, Lu et al^10^ found that K allele frequency was higher in CHD patients than in controls, and K allele carriers develop myocardial infarction more easily. In Egyptian patients with peripheral arterial occlusive disease, Shaker et al^11^ found a statistical difference in the clinical history and laboratory data between the EE and EK variants among those with ischemic heart disease.

A study by Banda et al^12^ showed that mice with *ICAM-1* deficiency had normal endothelial function (vasorelaxation in response to acetylcholine) after ischemia-reperfusion, whereas wild-type mice had impaired vasorelaxation in response to acetylcholine, indicating that *ICAM-1* gene function may be related to impaired endothelium-dependent vasodilatation. This dysfunction of the endothelium plays a key role in all stages of atherosclerosis.

On the other hand, McGlinchey et al^13^ found no association between the *ICAM-1* K469E polymorphism and CHD in a well-defined Irish population. Similarly, Aminian et al^14^ found no significant differences between CHD patients and controls as regards KK genotype and concluded that there was no strong relation between K469E polymorphisms and occurrence of CHD and MI in the studied population from Fars province, Iran. Also, a Slovenian study reported that the K469E polymorphism of the *ICAM-1* gene was not associated with MI in subjects with type 2 diabetes.^15^ The discrepancy among the studies could be due to selection of patient groups from different ethnic populations, variable sample sizes or different interactions of the genetic background with environmental factors.

In the present study, the frequencies of the K genotype (KK and EK) in acute MI patients (61.6%) showed no significant difference as compared to UA patients (44.4%) (*P*=.121). Previous studies compared ICAM-1 K469E polymorphisms between MI and CHD group as a whole.^7^,^14^ In our work, there were no significant differences in sICAM-1 levels between CHD patients and control subjects (*P*=.37) and no significant differences in sICAM-1 levels among patients with different genotypes (*P*=.53). However, sICAM-1 level elevation among patients with coronary disease was reported by Ridker et al,^16^ Malik et al^17^ and Güray et al.^18^ Also, in the Han population of China, individuals with the K allele had higher plasma level of sICAM-1 than those without the K allele.^10^ This difference in results may be due to the fact that most of the patients in the current study had an acute condition, while the patients in some of the previous studies had chronic CHD. In a prospective study, Albert et al^2^ found that among women without a history of cardiovascular disease, sICAM-1 levels were predictive of cardiovascular events that reflect coronary atherosclerotic disease progression and vessel narrowing as stable angina, but not those events associated with acute thrombosis/vessel occlusion as acute myocardial infarction. Discrepancies between sICAM-1 levels in different studies can be attributed to the racial/ ethnic differences in cardiovascular risk.^1^

The present study showed higher levels of sICAM-1 in men than women with CHD (*P*=.004). However, Zakai et al^19^ studied the relation between inflammation and cardiovascular disease risk (CVD) in older adults, and they found that sICAM-1 was associated with CVD in women only. This difference may be attributed to the limited number of female patients in our study or the different ages of patients. In comparison between UA patients and acute MI patients, there was no significant difference as regards the sICAM levels (*P*=.23); this agrees with the findings of Güray et al^18^ and Damnjanovic et al.^20^

In conclusion, our results suggest that the KK and EK genotypes of the *ICAM-1* gene polymorphism in codon 469 are associated with the risk for CHD development in Egyptians. Circulating sICAM-1 is not influenced by this polymorphism and is not necessarily elevated in acute coronary heart disease.
